# Explainable residual CNN with Gramian Angular Field encoding for rapid quantification of soil properties using near-infrared spectroscopy

**DOI:** 10.3389/fpls.2026.1953112

**Published:** 2026-10-14

**Authors:** Xiong Li, Junwei Guo, Ran Wan, Le Zhao, Zengping Chen

**Affiliations:** 1 Zhengzhou Tobacco Research Institute of CNTC, Zhengzhou, China; 2 College of Chemistry and Chemical Engineering, Hunan University, Changsha, China

**Keywords:** chemical composition of soil, Gramian Angular Field, output feature maps, residual connections, visual encoding

## Abstract

**Background:**

Soil chemical composition is a key indicator of soil fertility, yet conventional physicochemical analysis is time-consuming and costly. Although near-infrared (NIR) spectroscopy enables rapid, non-destructive detection, traditional linear models struggle with the non-linear nature of high-dimensional spectral data, and deep-learning models often suffer from a "black-box" problem.

**Methods:**

A one-dimensional residual convolutional neural network (1D-Res-CNN) was constructed, and the output feature maps of intermediate convolutional layers were visualized to reveal how the model learns spectral features. One-dimensional spectral sequences were mapped into twodimensional images via Gramian Angular Summation Field (GASF) and Continuous Wavelet Transform (CWT); after systematically comparing encoding performance across spectral sub-intervals, the optimal range was selected to feed a two-dimensional residual CNN (2D-Res-CNN) regression model. Using SNV-preprocessed Fourier-transform NIR spectra and an SPXY-partitioned dataset, the proposed framework was benchmarked against partial least squares regression (PLSR), k-nearest neighbors (KNN), and random forest (RF).

**Results:**

First-layer kernels in the 1D-Res-CNN automatically captured spectral features corresponding to functional-group absorption peaks, supporting the physical interpretability of feature extraction. GASF-encoded images in the 5880~4000 cm^-1^ range exhibited the highest feature density and outperformed CWT encoding. The 2D-Res-CNN achieved prediction-set $R_{p}^{2}$ of 0.87, 0.91, 0.91, 0.93, and 0.88 for soil pH, labile organic carbon, total nitrogen, organic matter, and available phosphorus, respectively, with lower RMSEP than both the 1D-CNN and PLSR.

**Conclusion:**

By enhancing spectral feature expression through visual encoding and providing preliminary insight into model behavior via layer-wise feature-map visualization, this work offers an interpretable technical strategy for rapid quantitative analysis of soil chemical composition by NIR spectroscopy.

## 1 Introduction

Crop growth and quality formation are directly governed by soil chemical composition. Key soil constituents—including pH, labile organic carbon, total nitrogen, organic matter, and available phosphorus—modulate the assimilation, transformation, and accumulation of mineral nutrients within plants, thereby exerting an indirect but decisive influence on agricultural yield and product quality (Ishfaq et al., 2023; Xia et al., 2025). For instance, soil pH directly regulates nutrient bioavailability; labile organic carbon serves as the primary energy and carbon source for soil microorganisms, driving their metabolic activity; total nitrogen is a fundamental element in the synthesis of proteins and nucleic acids; organic matter ameliorates soil physical structure and facilitates root extension; and phosphorus participates in core physiological processes such as energy transfer and photosynthesis (Lu et al., 2022; Wang et al., 2024). Consequently, rapid and accurate acquisition of soil chemical information is essential for precision agricultural management. Nevertheless, conventional physicochemical analysis necessitates labor-intensive pretreatments—including air-drying, grinding, digestion, and distillation—which are inherently time-consuming, costly, and poorly suited to high-throughput, real-time detection at the field scale (Chen et al., 2022).

Near-infrared spectroscopy has emerged as a prominent non-destructive technique for soil chemical analysis, owing to its low cost, rapid response, and suitability for online deployment (Shepherd and Walsh, 2002a). However, NIR spectral data are characterized by high dimensionality, severe multicollinearity, and nonlinear distortions induced by instrumental noise and scattering effects. Traditional chemometric models exhibit distinct mechanisms and limitations when applied to high-dimensional spectral data. Partial least squares regression (PLSR) relies on linear latent-variable decomposition, which may be insufficient for complex nonlinear spectral–constituent mappings. Least squares support vector machines (LS-SVM) can capture nonlinear relationships via kernel-induced feature mapping, yet their performance is sensitive to kernel selection and hyperparameter tuning. Random forests (RF) provide flexible nonlinear approximation through ensemble decision trees, but they often struggle with high-dimensional, strongly collinear spectral data and may suffer from overfitting when the feature-to-sample ratio is high. Consequently, these methods frequently exhibit limited capacity for robustly resolving intricate spectral–chemical constituent mappings in soil NIR analysis (Barra et al., 2021). In recent years, deep learning algorithms, particularly convolutional neural networks (CNNs), have demonstrated remarkable advantages in spectral quantitative tasks by hierarchically extracting nonlinear features through cascaded convolutional operations, as evidenced in moisture detection in agricultural products (Yang et al., 2024) and quality assessment of medicinal materials (Li et al., 2023). Yet, the internal decision-making mechanisms of CNNs have long been criticized as “black boxes”: although convolutional layers generate output feature maps through iterative convolutional operations, connecting multi-level nonlinear representations, existing studies rarely provide systematic exposition or mechanistic interpretation of the layer-wise feature learning trajectory (Cai et al., 2022). In the context of soil chemical detection, prioritizing predictive accuracy over model trustworthiness restricts the practical adoption of these methods in agricultural production. Therefore, visualizing intermediate-layer features to uncover the intrinsic logic of spectral feature extraction is imperative for enhancing model transparency and interpretability.

Deep one-dimensional CNNs with stacked convolutional layers, dilated kernels, or large receptive fields can, in principle, capture long-range spectral dependencies. However, such dependencies are learned implicitly through hierarchical feature abstraction, which typically demands substantial network depth and large training datasets to stabilize. In contrast, Gramian Angular Field encoding explicitly constructs a two-dimensional correlation matrix in which cross-band relationships are directly represented as spatial proximity and intensity patterns, allowing two-dimensional convolutional kernels to operate on these pre-computed structural relationships without requiring the network to learn them from scratch. Similarly, Continuous Wavelet Transform explicitly decouples the spectral signal into localized frequency components across scales, producing a two-dimensional scale–wavenumber representation that captures multi-resolution features in a mathematically rigorous manner. Therefore, the representational advantage of GAF and CWT lies not in enabling cross-band correlation modeling per se, but in providing an explicit, structurally preserved, and interpretable two-dimensional formulation of the intrinsic one-dimensional dependencies, thereby enhancing both the efficiency of feature extraction and the physical interpretability of the learned representations. Zhao et al. (Liu et al., 2025) converted spectral data into time-frequency spectrograms via continuous wavelet transform and achieved accurate determination of chlorophyll content in potato leaves. Tan et al. (2023) transformed near-infrared spectra of compound fertilizers into Gramian Angular Field (GAF) images, employing quaternion convolutional neural networks to significantly improve the precision of simultaneous multi-component detection. Wang et al. (2022) introduced Markov Transition Field (MTF) images to enhance the predictive stability of aflatoxin B1 in maize. Among these approaches, GAF maps one-dimensional sequences into symmetric Gram matrices via a cosine kernel, preserving the dependency and structural integrity of the original spectral data while achieving two-dimensional image encoding. This property renders GAF particularly suitable for near-infrared spectral analysis, which exhibits strong sequential correlations, compared with CWT and MTF (Si et al., 2023). Previous studies have coupled GAF with deep learning for quantitative analysis of laser-induced fluorescence spectra (Si et al., 2023), alcohol-diesel mixtures (Liu et al., 2021), and moldy peanuts (Jiang et al., 2023), validating the efficacy of this strategy. However, existing literature has predominantly focused on the isolated application of either GAF or CWT, without systematically comparing their feature representation capabilities across different spectral sub-intervals. Moreover, most studies have targeted single chemical constituents, leaving the applicability of GAF-encoded residual CNNs to simultaneous soil (chemical) properties prediction in soil largely unexplored. Furthermore, the lack of organic integration between 1D-CNN feature visualization and 2D-CNN visual encoding prevents the formation of a complete technical chain from mechanistic interpretation to predictive enhancement.

Against this backdrop, the present study proposes an interpretable deep-learning framework that couples visual encoding with residual-connected CNNs for soil chemical detection, aiming to simultaneously improve predictive accuracy and visualize internal model mechanisms. The specific objectives are: (1) To construct a one-dimensional residual CNN (1D-Res-CNN) and systematically elucidate its feature-learning trajectory in the spectral analytic context through intermediate-layer feature-map visualization, offering qualitative insight suggestive of physically meaningful spectral feature extraction; (2) To introduce GASF and CWT for mapping one-dimensional spectral sequences into two-dimensional texture images, compare their encoding performance across spectral sub-intervals, select the optimal range, and establish a two-dimensional residual CNN (2D-Res-CNN) regression model for systematic benchmarking of the two visual encoding paradigms; and (3) To validate the proposed framework against PLSR, KNN, and RF benchmarks for the quantification of soil pH, labile organic carbon, total nitrogen, organic matter, and available phosphorus, thereby furnishing a novel technical strategy for rapid, accurate, and interpretable detection of soil chemical composition.

## 2 Materials and methods

### 2.1 Sample preparation and experimental framework

Soil samples were collected from agricultural fields under active crop cultivation in Huidong County, Sichuan Province, China (approximately 26.6°N, 102.6°E), located in the southwestern mountainous region of the country. The regional climate is subtropical monsoon, with a mean annual temperature of 16.2^。^C and mean annual precipitation of 1,050 mm. The dominant soil types in this region are Purplish soils (Regosols) and Red soils (Ferralsols) according to the Chinese Soil Taxonomy, developed primarily from purple sandstone and mudstone parent materials. The fields were under continuous crop cultivation, predominantly involving a tobacco-maize rotation system. The sampling campaign covered 45 regions within the county, and 5~7 agricultural fields were selected in each region, yielding a total of 301 fields. Within each field, surface soil (0~20 cm depth) was collected at 8 sampling points distributed across different locations of the field using a stainless-steel auger. No compositing was performed: each sampling point was collected and bagged independently, constituting one independent physical sample, resulting in 301×8 = 2,408 physical samples in total. All samples were air-dried under ambient conditions for 14 days, gently crushed, and passed through a 2 mm sieve; a representative subsample of each physical sample was further ground to<0.25 mm using a planetary ball mill. Diffuse reflectance spectra were acquired using a Bruker TENSOR II FT-NIR spectrometer (Bruker Optics, Ettlingen, Germany) over the 10,000~4,000 cm^-1^ range (resolution 8 cm^-1^; sampling interval 1.928 cm^-1^; 3,001 data points per spectrum). Each sample was scanned 64 times, and the 64 scans were co-added and averaged by the instrument to yield one spectrum per physical sample; thus, the number of spectral observations equals the number of physical samples (2,408). The raw reflectance was converted to pseudo-absorbance as log(1/R). Due to missing reference values or the exclusion of outliers during quality control, the effective sample sizes varied slightly among properties: pH (n = 2,195), labile organic carbon (n = 1,764), total nitrogen (n = 2,195), organic matter (n = 2,162), and available phosphorus (n = 2,195).

Rference measurements for the five chemical constituents followed standardized soil analytical protocols: Soil pH: Determined potentiometrically in a 1:5 soil-to-water suspension using a calibrated pH meter, following the protocol of the Soil Science Society of China (Kabała et al., 2016). Labile organic carbon:Measured by oxidation with 0.333 M KMnO_4_ followed by colorimetric determination at 565 nm (Blair et al., 1995). Total nitrogen: Determined by the Dumas dry combustion method using an elemental analyzer (Food and agriculture organization of the united nations, 2021). Organic matter was quantified by the Walkley–Black dichromate oxidation method: 1.000 g of air-dried soil was digested with 10 mL of 0.167 M K_2_Cr_2_O_7_ in concentrated H_2_SO_4_, the excess dichromate was back-titrated with 0.5 M FeSO_4_·(NH_4_)_2_SO_4_, and oxidizable organic carbon was calculated as OC (g kg^-1^) = [(V_blank−V_sample)×M_Fe×0.003×100×1.3]/m_soil, where M_Fe is the molarity of the ferrous solution and 1.3 is the conventional recovery-correction factor (Walkley and Black, 1934). Organic matter content was estimated from oxidizable organic carbon using the conventional conversion factor of 1.724. Available phosphorus: Extracted with 0.5 M sodium bicarbonate (NaHCO_3_, pH 8.5) following the Olsen method, and measured by the molybdenum-blue colorimetric method at 880 nm using a UV-visible spectrophotometer (Olsen et al., 1954). Due to occasional missing values or outlier exclusion during quality control (e.g., spectral leverage outliers or chemical residual outliers), the effective sample sizes varied slightly among properties: pH (n = 2,195), labile organic carbon (n = 1,764), total nitrogen (n = 2,195), organic matter (n = 2,162), and available phosphorus (n = 2,195).

The experimental workflow was organized into two stages (**Figure 1**). In Stage I, one-dimensional spectral modeling and interpretables 1D-Res-CNN analysis were performed. Raw spectra were preprocessed with multiplicative scatter correction (MSC), standard normal variate (SNV), and Savitzky–Golay (SG) smoothing. PLSR, KNN, RF、and 1D-Res-CNN models were subsequently constructed. Model performance was evaluated via cross-validation using the coefficient of determination (R^2^) and root-mean-square error (RMSE), enabling selection of the optimal preprocessing strategy and baseline model. The output feature maps of the three convolutional layers in the 1D-Res-CNN were then visualized to elucidate the model’s spectral feature-learning mechanisms. In Stage II, visual encoding and two-dimensional modeling were conducted. The full spectrum was partitioned into three sub-intervals: 5880~4000 cm^-1^, 10000~5880 cm^-1^, and 10000~4000 cm^-1^. Within each interval, one-dimensional spectral sequences were converted into two-dimensional images via Gramian Angular Field and Continuous Wavelet Transform (CWT). These encoded images were fed into a 2D-Res-CNN for quantitative prediction of soil chemical constituents. The predictive performance of different encoding methods and spectral intervals was systematically assessed using R^2^ and RMSE, thereby identifying the optimal visual encoding strategy.

**Figure 1:**
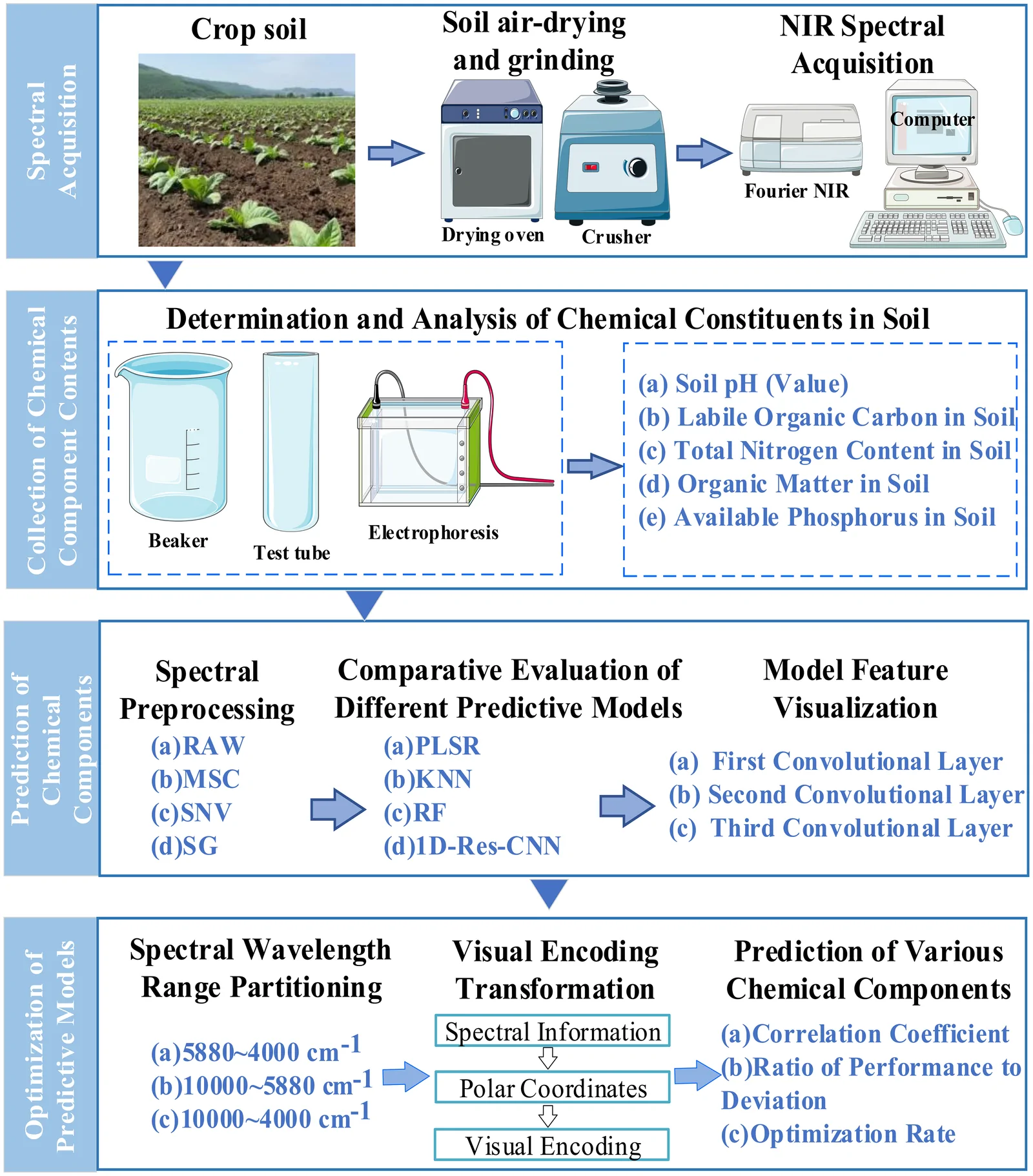
Key steps of the experimental procedure.

### 2.2 Spectral preprocessing and dataset partitioning

Spectral preprocessing was performed to mitigate interference from scattering effects, baseline drift, and high-frequency noise. Three strategies—MSC, SNV, and SG smoothing—were systematically compared. Spectral preprocessing was performed using custom scripts in Python 3.9 with NumPy 1.23 and scikit-learn 1.2. The exact settings were as follows:MSC: Linear correction against the mean reference spectrum of the calibration set.SNV: Per-spectrum mean-centering and scaling to unit variance.SG smoothing: A Savitzky–Golay filter with a window length of 15 points, a 2nd-order polynomial, and 0th derivative (smoothing only) was applied. For derivative-enhanced peak detection, a 2nd derivative with the same window and polynomial order was also evaluated but not selected as optimal (Mishra and Lohumi, 2021). The preprocessed spectra were subsequently used to construct PLSR, KNN, and 1D-Res-CNN models, and the optimal preprocessing strategy was selected through cross-validation for subsequent one-dimensional modeling and two-dimensional visual encoding analysis.

This study constructed five independent single-output regression models, each model was trained and evaluated using a property-specific SPXY partition of the dataset, the 1D-Res-CNN and 2D-Res-CNN architectures both employ a single linear output neuron for regression prediction of one target property at a time. To ensure an unbiased estimate of predictive performance, a rigorous validation framework was implemented. The entire dataset was first partitioned into a calibration set (75%) and a prediction set (25%) using the SPXY algorithm. This prediction set was reserved as a truly untouched test set and was not accessed during any model-selection procedures. All subsequent comparisons, including preprocessing methods, spectral interval selection, encoding strategies and hyperparameter optimization, were conducted exclusively within the calibration set using nested cross-validation with a 5-fold outer loop, in which hyperparameter tuning (e.g., the number of PLSR latent variables) was performed in an inner loop using 10-fold cross-validation on the inner calibration data only. In the nested structure, the outer loop provided unbiased performance estimates for comparing different methodological choices, while the inner loop was used solely for hyperparameter tuning and for selecting the optimal number of latent variables in PLSR and the optimal k in KNN. The final reported metrics were calculated only once on the untouched prediction set, using the final model configuration determined from the nested cross-validation within the calibration set. All replicate scans from the same physical sample were retained in the same fold during cross-validation. This prevents spatial autocorrelation leakage and ensures that the validation reflects the model’s generalization capability to new, unseen fields.

### 2.3 Visual encoding strategies

Near-infrared spectral data are intrinsically one-dimensional signals with strong sequential correlations, whereas 2D-Res-CNN require spatially structured inputs. To bridge this dimensional gap, this study employs GASF and CWT to recast one-dimensional spectral sequences into two-dimensional texture images, thereby unlocking the feature-extraction potential of 2D-Res-CNN. The advantage over 1D-Res-CNN: While 1D-Res-CNN capture local sequential patterns, they do not explicitly construct cross-band correlation matrices. GASF and CWT restructure these implicit relational and multi-scale features into a two-dimensional format, allowing 2D convolutional kernels to operate directly on pre-computed structural relationships. To ensure a rigorous and equitable comparison, both encoding strategies were applied to identical spectral sub-intervals, and the resulting two-dimensional images were resized to uniform pixel dimensions prior to 1D-Res-CNN training.

#### 2.3.1 Gramian angular field

GAF is not a spatial injection but a structural re-expression: The Gramian Angular Field generates a symmetric Gram matrix where each element quantifies the cosine-weighted angular relationship between a pair of spectral positions. This explicitly encodes the global dependency and pairwise correlation structure inherently present in the one-dimensional spectral sequence, rather than inventing non-existent spatial dimensions. GAF can be divided into Gramian Angular Summation Field GASF and Gramian Angular Difference Field. Among these, GASF demonstrates superior spectral characterization by computing a cosine-weighted summation of wavenumber (cm^-1^) sequences, thereby preserving both magnitude and phase information more effectively than GADF’s difference operation (Gao et al., 2022). The Gramian Angular Summation Field was employed. The spectral sequence was first Min-Max normalized to [−1, 1], then transformed into polar coordinates. The GASF matrix was computed as GASF, yielding a symmetric *N*×*N* matrix. For computational efficiency, the matrix was resized to 224×224 pixels via bicubic interpolation, producing a single-channel grayscale image in the range [−1, +1]. A sensitivity analysis confirmed that resizing to 128×128, 224×224, and 256×256 yielded comparable predictive performance, indicating minimal information loss at the selected resolution. The steps of GASF process to realize the output of 2D image are as follows (Equation 1):

(1) $${\tilde{x}}_{i}=\frac{x_{i}-Min(x)}{Max(x)-Min(x)}\times 2-1$$

The spectral sequence in the Cartesian coordinate system is transformed into polar coordinates with the formula shown in (Equation 2). where *ϕ* is the angle and *r* is the polar diameter. $r_{i}=\frac{i}{m},\;i\in m$, *i* represents the wavenumber (cm^-1^) index and *m* represents the wavenumber (cm^-1^).

(2) $$\phi_{i}=\arccos({\tilde{x}}_{i}),-1\leq \tilde{x}\leq 1,\;{\tilde{x}}_{i}\in \tilde{x}$$

(Equations 3, 5) are the definitions of the Gram angle sum field and the Gram angle difference field, respectively. The size of the visually encoded image depends on the number of original spectral bands, and the size is *m*×*m*. Equations 4 and 6 are the definitions of GASF and GADF.

(3) $$GASF=\left[\begin{array}{c} \cos\left(\phi_{i}+\phi_{j}\right) \end{array}\right]=\left[\begin{array}{cccc} \cos\left(\phi_{1}+\phi_{1}\right) & \cos\left(\phi_{1}+\phi_{2}\right) & \cdots & \cos\left(\phi_{1}+\phi_{m}\right)\\ \cos\left(\phi_{2}+\phi_{1}\right) & \cos\left(\phi_{2}+\phi_{2}\right) & \cdots & \cos\left(\phi_{2}+\phi_{m}\right)\\ \vdots & \vdots & \vdots & \vdots \\ \cos\left(\phi_{m}+\phi_{1}\right) & \cos\left(\phi_{m}+\phi_{2}\right) & \cdots & \cos\left(\phi_{m}+\phi_{m}\right) \end{array}\right]$$

(4) $$={\tilde{x}}^{T}\tilde{x}-{\sqrt{I-{\tilde{x}}^{2}}}^{T}\sqrt{I-{\tilde{x}}^{2}}$$

(5) $$GADF=\left[\begin{array}{c} \sin\left(\phi_{i}-\phi_{j}\right) \end{array}\right]=\left[\begin{array}{cccc} \sin\left(\phi_{1}-\phi_{1}\right) & \sin\left(\phi_{1}-\phi_{2}\right) & \cdots & \sin\left(\phi_{1}-\phi_{m}\right)\\ \sin\left(\phi_{2}-\phi_{1}\right) & \sin\left(\phi_{2}-\phi_{2}\right) & \cdots & \sin\left(\phi_{2}-\phi_{m}\right)\\ \vdots & \vdots & \vdots & \vdots \\ \sin\left(\phi_{m}-\phi_{1}\right) & \sin\left(\phi_{m}-\phi_{2}\right) & \cdots & \sin\left(\phi_{m}-\phi_{m}\right) \end{array}\right]$$

(6) $$=\sqrt{I-{\tilde{x}}^{2}}\tilde{x}-{\tilde{X}}^{T}\sqrt{I-{\tilde{x}}^{2}}$$

#### 2.3.2 Continuous wavelet transform

CWT is a standard multi-scale signal decomposition: The Continuous Wavelet Transform produces a two-dimensional scale-wavenumber coefficient matrix that captures localized frequency characteristics across scales. This is a well-established scale-wavenumber analysis technique that re-expresses intrinsic signal structure. The Continuous Wavelet Transform was performed using the Morlet mother wavelet with 64 scales logarithmically spaced from 1 to 128. The wavelet coefficient matrix *w*(*a*, *b*) was computed via the PyWavelets package, normalized to [0, 1] by linear scaling, and resized to 224×224 pixels via bicubic interpolation to generate a single-channel 2D spectral map. This study employs the CWT to perform joint scale-wavenumber analysis of the original spectral signals, thereby constructing two-dimensional NIR spectral maps. Let the original one-dimensional NIR spectrum be denoted as.

(7) x(λ), λ=1,2,…,N

where λ represents the wavenumber (cm^-1^) index and Ndenotes the number of spectral bands. The CWT is defined as the inner product between the signal and a mother wavelet function ψ(.) under different scale parameters and translation parameters b, and can be mathematically expressed as.

(8) $$W\left(a,b\right)=\frac{1}{\sqrt{\left|a\right|}}\int_{-\infty }^{+\infty }x\left(\lambda \right)\psi^{*}\left(\frac{\lambda -b}{a}\right)d\lambda$$

where a>0 is the scale factor that controls the dilation of the wavelet to characterize features at different frequencies, bis the translation parameter corresponding to the local position along the wavenumber (cm^-1^) axis, and ψ∗(.) denotes the complex conjugate of the mother wavelet. By computing the wavelet coefficients W(a,b) over multiple scales and wavenumber (cm^-1^) positions, a two-dimensional coefficient matrix is obtained, in which the horizontal axis corresponds to the wavenumber (cm^-1^) position band the vertical axis corresponds to the scale parameter a, thereby forming a two-dimensional NIR spectral map. This two-dimensional representation simultaneously captures local spectral variations across multiple scales, enabling a more comprehensive characterization of spectral features than the original one-dimensional signal and facilitating the extraction of richer discriminative information for subsequent analysis.

### 2.4 Deep learning architectures

To accommodate the dimensional characteristics of spectral data, this study constructed two residual-connected convolutional neural networks: 1D-Res-CNN and a 2D-Res-CNN (**Figure 2**), for one-dimensional spectral feature visualization and two-dimensional image-based regression prediction, respectively. The architectural hyperparameters of both networks were determined via random search within the inner loop of the nested cross-validation conducted exclusively within the calibration set. The search space comprised kernel sizes of {1×3, 1×5, 1×7} for the 1D-Res-CNN and {3×3, 5×5} for the 2D-Res-CNN, filter numbers of {8, 16, 32, 64}, learning rates of {0.001, 0.0005, 0.0001}, and batch sizes of {16, 32, 64}. The configuration yielding the lowest RMSECV was selected as the final architecture, ensuring that all parameter tuning was strictly confined to the calibration data.

**Figure 2:**
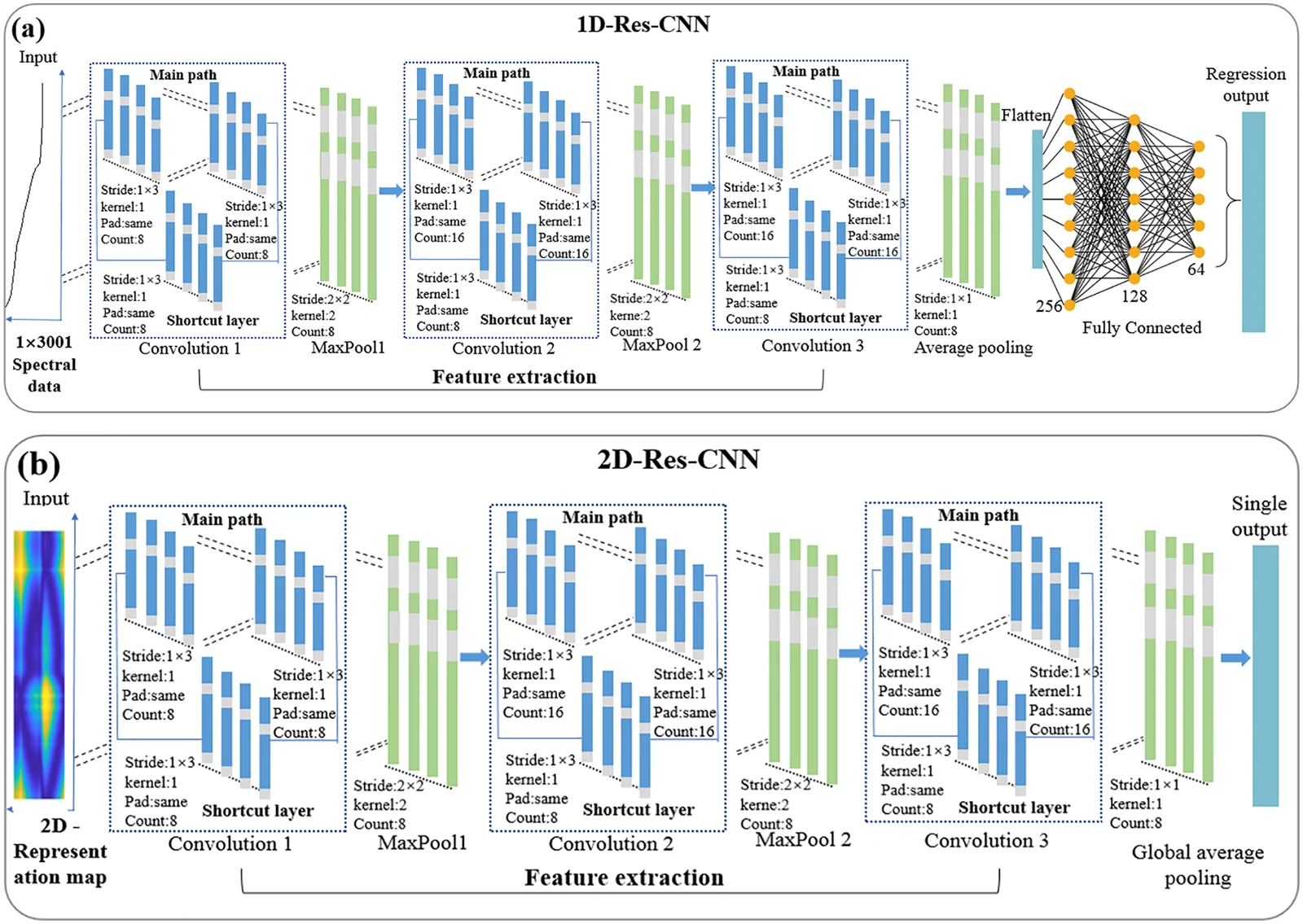
**(a)** Model framework of the 1D-Res-CNN; **(b)** 2D-Res-CNN.

#### 2.4.1 1D-Res-CNN with residual connections for feature visualization

The 1D-Res-CNN accepts an input dimension of 1×3001, corresponding to the full spectral band count (Zhang et al., 2020; Dabas et al., 2025). The network comprises three convolutional layers, three pooling layers (incorporating both average and max pooling), and a fully connected regression module. Each convolutional layer consists of a main pathway and a shortcut layer forming a residual block: the main pathway is responsible for feature extraction and transformation, while the shortcut layer employs an identity mapping to mitigate gradient vanishing and enhance feature propagation (ablation experiments showed that removing residual connections slowed convergence and decreased the prediction-set $R_{p}^{2}$ by 3~5%). The first convolutional layer focuses on extracting elementary spectral features, whereas the second and third layers progressively learn more abstract composite representations. Pooling operations reduce spatial dimensionality while preserving salient activations. Following the third pooling layer, the refined features are flattened and fed into a three-layer fully connected network with 256, 128, and 64 neurons, respectively. A final single-neuron linear output layer produces the predicted chemical constituent concentrations.

To demystify the “black box” of the model, output feature maps from all three convolutional layers were extracted and visualized. The first layer yields 16 feature maps, revealing the initial response of convolutional kernels to individual spectral bands; the second layer produces 32 feature maps, reflecting the feature abstraction process; and the third layer generates 64 feature maps, presenting deep semantic activations. By spatially aligning activation peaks with known functional-group absorption bands, the physical interpretability of 1D-Res-CNN-driven feature extraction was validated.

#### 2.4.2 2D-Res-CNN for spectrogram regression

The 2D-Res-CNN takes two-dimensional spectrograms generated by GASF or CWT as input. Compared with its one-dimensional counterpart, the 2D residual network simultaneously captures local spectral details and global structural patterns, extracting joint spatial-spectral features through two-dimensional convolutional kernels. The residual connection mechanism enables the construction of deeper network hierarchies, ensuring stable gradient propagation during training and improving both convergence speed and model robustness. The network terminus employs global average pooling in lieu of fully connected layers to reduce parameter count and suppress overfitting (ablation experiments indicated that replacing global average pooling with a 256-neuron fully connected layer increased the parameter count by approximately 40% without improving prediction accuracy), followed by a single-neuron linear output layer for regression prediction of soil chemical constituent concentrations (Zhong et al., 2021). The detailed structural parameters of the 1D-Res-CNN are summarized in **Table 1**. Notably, the 1D-Res-CNN contains approximately 0.45 M parameters with a computational cost of 12.6 MFLOPs per inference, whereas the 2D-Res-CNN contains approximately 0.38 M parameters and ~9.8 MFLOPs; the comparable—and in fact slightly lower—capacity of the 2D model indicates that the observed performance improvement is attributable to the visual encoding strategy rather than to an inflation of network complexity. The detailed architectural configurations of both networks, including input/output dimensions, kernel sizes, filter numbers, strides, padding, activation functions, normalization, pooling, dropout, residual connections, and trainable parameters, are comprehensively summarized in **Table 1**.

**Table 1:** Architectural specifications of 1D-Res-CNN and 2D-Res-CNN.

(a) 1D-Res-CNN						
Layer	Kernel size	Stride	Padding	Output channels	Output shape	Activation
Input	—	—	—	1	1 × 3001	—
Conv-1 (main)	1 × 3	1	Same	16	16× 3001	ReLU
Conv-1 (shortcut)	1 × 1	1	Same	16	16× 3001	ReLU
MaxPool-1	1 × 2	2	—	16	16 × 1500	—
Conv-2 (main)	1 × 3	1	Same	32	32 × 1500	ReLU
Conv-2 (shortcut)	1 × 1	1	Same	32	32 × 1500	ReLU
MaxPool-2	1 × 2	2	—	32	32× 1500	—
Conv-3 (main)	1 × 3	1	Same	64	64× 750	ReLU
Conv-3 (shortcut)	1 × 1	1	Same	64	64× 750	ReLU
Average pooling	1 × 1	1	—	64	64 × 375	—
Flatten	—	—	—	—	2400	—
FC-1	—	—	—	—	256	ReLU
Dropout	—	—	—	—	256	—
FC-2	—	—	—	—	128	ReLU
FC-3	—	—	—	—	34	ReLU
Output	—	—	—	—	1	Linear
(b) 2D-Res-CNN						
Layer	Kernel size	Stride	Padding	Output channels	Output shape	Activation
Input	—	—	—	1	1 × 224 × 224	—
Conv-1 (main)	3 × 3	1	Same	16	16 × 224 × 224	ReLU
Conv-1 (shortcut)	3 × 3	1	Same	16	16 × 224 × 224	ReLU
MaxPool-1	2 × 2	2	—	16	16 × 122 × 122	—
Conv-2 (main)	3 × 3	1	Same	32	32 × 122 × 122	ReLU
Conv-2 (shortcut)	1 × 1	1	Same	32	32 × 122 × 122	ReLU
MaxPool-2	2 × 2	2	—	32	32 × 56 × 56	—
Conv-3 (main)	3 × 3	1	Same	64	64 × 56 × 56	ReLU
Conv-3 (shortcut)	1 × 1	1	Same	64	64 × 56 × 56	ReLU
Global average pooling	—	—	—	64	64	—
Output	—	—	—	—	1	Linear

### 2.5 Benchmark models and evaluation metrics

PLSR integrates principal-component-based dimensionality reduction with multiple linear regression by constructing orthogonal latent variables that maximize the covariance between the spectral data matrix X and the chemical constituent concentration vector y. The high-dimensional collinear spectra are projected into a parsimonious latent space, after which a linear regression is fitted between the latent variables and each target constituent, rendering PLSR a robust linear baseline for high-dimensional spectroscopic modeling. KNN is an instance-based non-parametric learning method that retrieves the K closest training samples to a query point via a predefined distance metric and aggregates their chemical concentrations—typically by arithmetic averaging—to yield the predicted value, serving as a shallow nonlinear benchmark. Random Forest (RF) constructs an ensemble of multiple decorrelated decision trees via bootstrap aggregating (bagging) and random feature subset selection at each node split; the final prediction is obtained by averaging the outputs of all individual trees, thereby enhancing generalization and suppressing overfitting. PLSR: The optimal number of latent variables was determined by 10-fold cross-validation within the inner loop of the nested scheme (i.e., using the inner calibration data only), with candidate LVs ranging from 1 to 20. KNN: A grid search was performed within the calibration set over k∈{1, 3, 5, 7, 9, 11, 13, 15}, distance metrics, and weighting schemes. The configuration minimizing RMSECV was selected. RF: Bayesian optimization was applied to tune the number of trees and maximum depth.

All models were trained and evaluated on identical calibration and prediction subsets partitioned by the SPXY algorithm to ensure consistency of comparative conditions.

For clarity, the experimental conditions of every model are explicitly specified as follows. PLSR, KNN, RF, and the 1D-Res-CNN were applied to SNV-preprocessed spectra over the full spectral range (10,000~4,000 cm^-1^), whereas the GAF-Res-CNN and CWT-Res-CNN were applied to raw spectra (i.e., without any preprocessing) over all three spectral intervals (10,000~4,000, 5880~4,000, and 10,000~5880 cm^-1^). For each soil property, all models were trained and evaluated on identical samples using the same property-specific SPXY partitions, ensuring that any performance difference originates from the modeling strategy or the encoding approach rather than from differences in samples, partitions, or spectral range. Model performance was comprehensively assessed using five complementary metrics. The root-mean-square error of cross-validation (RMSECV) captures the stability of prediction during k-fold cross-validation on the calibration set, whereas the root-mean-square error of prediction (RMSEP) quantifies the absolute error on the independent test set; smaller values of both signify higher predictive accuracy. The coefficients of determination for cross-validation ( $R_{cv}^{2}$) and for prediction ( $R_{p}^{2}$) measure the proportion of variance in the target variable explained by the model on the calibration and test sets, respectively, with values approaching 1 indicating excellent goodness-of-fit. The mathematical formulations are as follows (Equations 9, 10):

(9) $$RMSE=\sqrt{\frac{1}{n}\sum_{i=1}^{n}{\left(y_{i}-{\tilde{y}}_{i}\right)}^{2}}$$

(10) $$R^{2}=1-\frac{\sum_{i=1}^{n}{\left(y_{i}-{\tilde{y}}_{i}\right)}^{2}}{\sum_{i=1}^{n}{\left(y_{i}-{\bar{y}}_{i}\right)}^{2}}$$

## 3 Result and discussion

### 3.1 Statistical analysis and spectral characteristics

Violin plots, which integrate the features of boxplots and kernel density estimations, provide an intuitive visualization of the probability density distribution of the normalized soil chemical constituent concentrations (Hintze and Nelson, 1998). **Table 2** presents the descriptive statistics of the contents of five chemical components in soil. Each violin plot corresponds to the distribution of a specific chemical constituent within the entire soil sample set. The overlaid scatter points highlight the concentration and spread of individual observations, enabling clear visual assessment of spectral trends across different wavenumber (cm^-1^) bands and their potential associations with specific soil chemical properties. This visualization is particularly effective for discerning variations in pH, labile organic carbon, total nitrogen, organic matter, and available phosphorus among different soil samples. **Figure 3** presents the normalized concentration distributions of the five soil chemical constituents. The violin plots reveal that pH values are predominantly distributed between 0.6 and 0.8, labile organic carbon between 0.5 and 0.7, total nitrogen and organic matter mainly in the 0.1~0.3 range, and available phosphorus in the 0.0~0.2 range. The width of each violin at specific levels reflects the density of observations, indicating the relative variability of each chemical property across the sampled soils. Moreover, the width and prominence of the violin plot at specific concentration levels reflect the density of observations within those ranges. A broader waist or pronounced peak indicates a higher concentration of sample values around that level, implying a dominant concentration range and lower relative variability for the corresponding chemical constituent. Conversely, a narrow or skewed distribution may suggest high variability or uneven distribution of that constituent across the sampled soils, possibly arising from heterogeneous soil composition or differing field management conditions.

**Table 2:** Descriptive statistics of the content of five chemical components in the soil.

Components	Entire dataset			Training dataset			Test dataset			P-value
	Mean	SD	CV (%)	Mean	SD	CV (%)	Mean	SD	CV (%)	
pH	6.62	1.12	16.90	6.63	1.11	16.74	6.61	1.13	17.09	0.17
Labile organic carbon	778.31	231.38	29.73	773.23	229.88	29.16	780.69	232.09	29.27	0.99
Total nitrogen	1.11	0.62	55.91	1.12	0.62	55.35	1.12	0.63	56.25	0.39
Organic matter	22.91	10.94	47.74	23.06	10.82	46.92	22.86	11.02	48.12	0.51
Available phosphorus	41.84	32.75	78.29	41.56	32.62	78.36	40.52	31.89	78.70	0.98

N denotes the total number of samples after quality control. Units: pH (dimensionless); labile organic carbon (mg·kg^-1^); total nitrogen (g·kg^-1^); organic matter (g·kg^-1^); available phosphorus (mg·kg^-1^). Sample sizes: pH (N = 2,195); labile organic carbon (N = 1,764); total nitrogen (N = 2,195); organic matter (N = 2,162); available phosphorus (N = 2,195).

**Figure 3:**
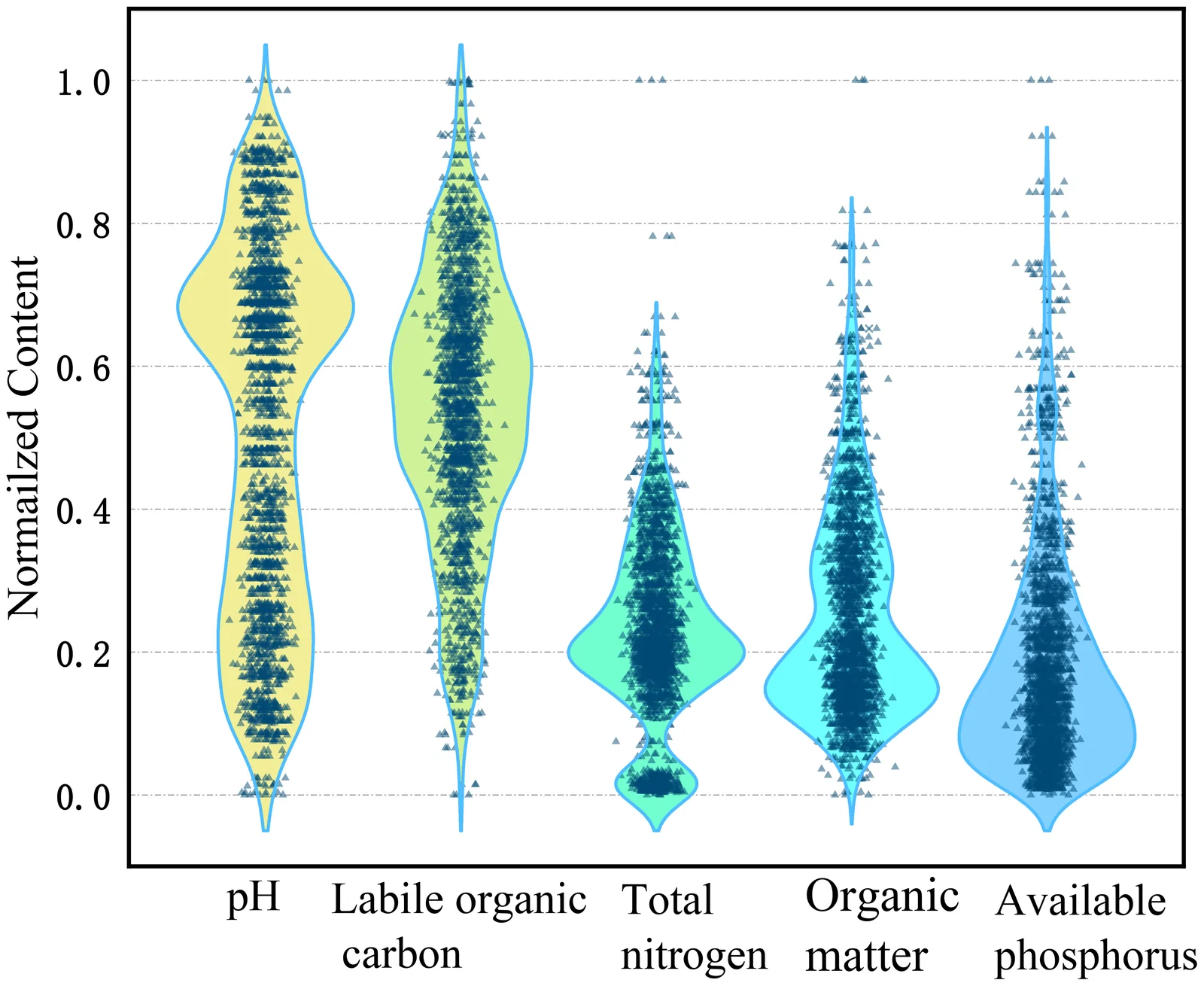
Violin plots and scatter plots of levels of chemical composition content.

### 3.2 Spectral characteristics

Representative spectra provide a clear depiction of the overall shape of the spectral curve, including absorption features, absorbance peaks, and other characteristic patterns within specific wavenumber (cm^-1^) regions, all of which are closely linked to the chemical composition and properties of field-scale agricultural soil (Viscarra Rossel et al., 2006). **Figure 4** presents the representative near-infrared absorption spectra of field-scale agricultural soils within the range of 10,000~4,000 cm^-1^. As shown in (**Figure 4a**), the spectral profiles reveal distinct differences in absorbance across seven specific spectral intervals. These characteristic peaks correspond to the inherent chemical signatures of the soil and reflect variations in internal chemical composition. Such spectral features can be leveraged for quantitative assessment and tracking of soil chemical constituents. The spectral profiles reveal distinct absorbance features across seven specific intervals (**Figure 4a**). The bands at 7268 cm^-1^ and 7070 cm^-1^ are consistent with the first overtone of O-H stretching vibrations, commonly associated with hydroxyl groups in soil organic matter and clay minerals (Workman and Weyer, 2007). The feature at 5216 cm^-1^ may potentially relate to the combination of C=O and O-H stretching within carboxyl groups found in humic substances (Shepherd and Walsh, 2002a). The regions at 5370 cm^-1^ and 4850 cm^-1^ show absorbance patterns associated with N-H bending and C-N stretching modes, which are statistically linked to total nitrogen in agricultural soils (Viscarra Rossel et al., 2006). The band near 4530 cm^-1^ has been indirectly associated with available phosphorus in previous soil spectroscopic studies, possibly reflecting P-O vibrational modes in phosphate structures or covariation with organic matter (Shepherd and Walsh, 2002b). These assignments represent indirect statistical associations rather than direct, unambiguous molecular identifications, given the complex overlapping nature of NIR overtone and combination bands in whole soils.

**Figure 4:**
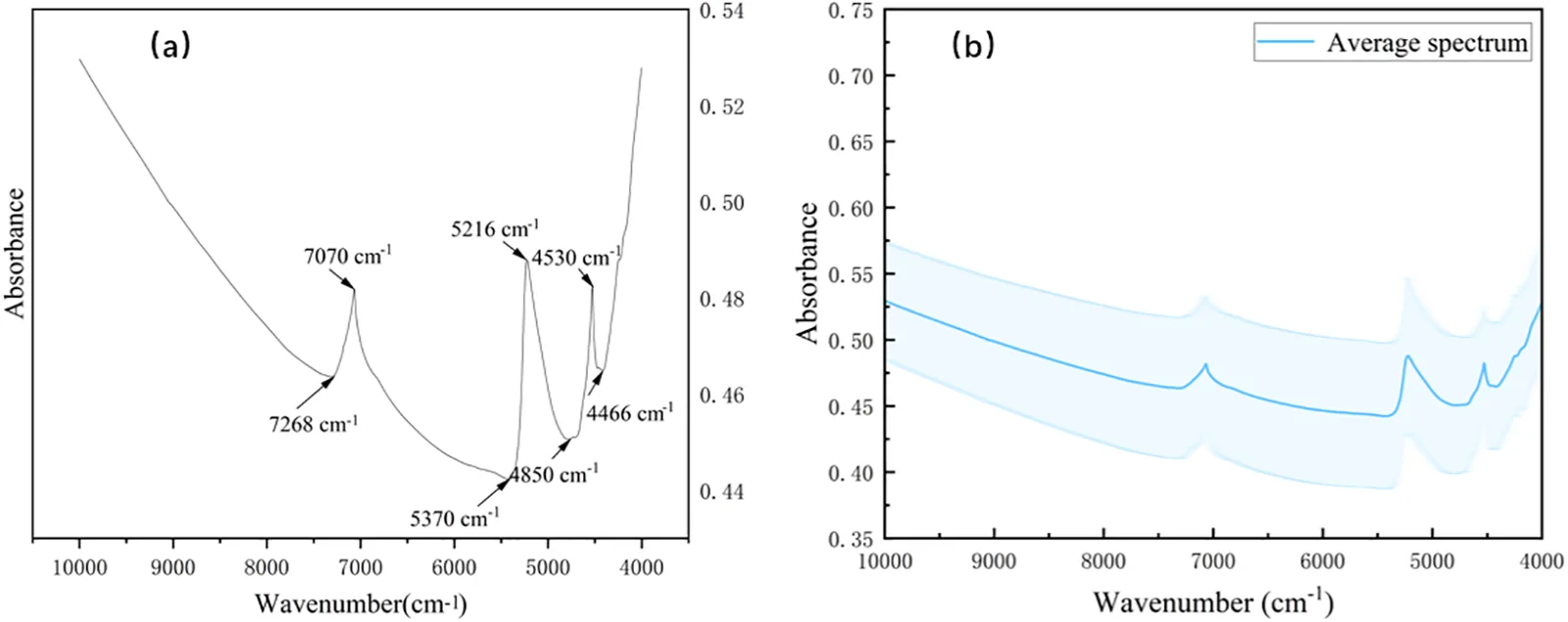
**(a)** Representative spectra; **(b)** Average spectra with error bands.

**Figure 4b** illustrates the mean spectrum with the shaded region representing the standard deviation among sample spectra (i.e., inter-sample variability). This variation reflects physicochemical heterogeneity among the collected soil samples rather than measurement error or instrumental precision. The pronounced fluctuations in the 7000~5000 cm^-1^ range indicate that this region is particularly sensitive to compositional differences among samples. To assess measurement repeatability independently, approximately 10% of samples were scanned in triplicate; the average within-sample relative standard deviation was<2.5%, confirming high instrumental repeatability. These results support the suitability of the spectral data as input for predictive modelling and provide a reliable basis for selecting informative wavenumber (cm^-1^) regions. Moreover, they highlight the potential of near-infrared spectroscopy in resolving complex chemical composition variations in soil (Viscarra Rossel et al., 2022).

### 3.3 Comparative analysis of preprocessing methods

To evaluate how spectral preprocessing influences NIR modelling, we compared the raw spectra with spectra processed by MSC, SNV, and SG filtering. **Figure 5** Correlation heatmaps between spectral wavenumber (cm^-1^) under different preprocessing strategies: (a) raw spectra, (b) MSC, (c) SNV, and (d) SG. The color intensity represents the Pearson correlation coefficient, as indicated by the color bar. Brighter regions denote stronger correlations, revealing the enhanced spectral–chemical associations achieved by SNV preprocessing. The raw spectra exhibit a distinct striped structure, indicating appreciable but uneven correlations. MSC largely homogenises baseline variation, yielding a generally brighter map. SNV, however, produces a darker, more intricately textured pattern across the spectrum, revealing the highest overall correlation intensity. SG enhances only specific regions—most prominently near 9,000 cm^-1^ and 7,000 cm^-1^, while offering limited improvement elsewhere. Overall, all three preprocessing methods improve the spectral-chemical associations relative to the raw data. SNV preprocessing produces a more intricate correlation structure with stronger off-diagonal associations compared to raw spectra, indicating that SNV enhances the intrinsic spectral–chemical relationships by suppressing scattering-induced baseline variations (Barnes et al., 1989).

**Figure 5:**
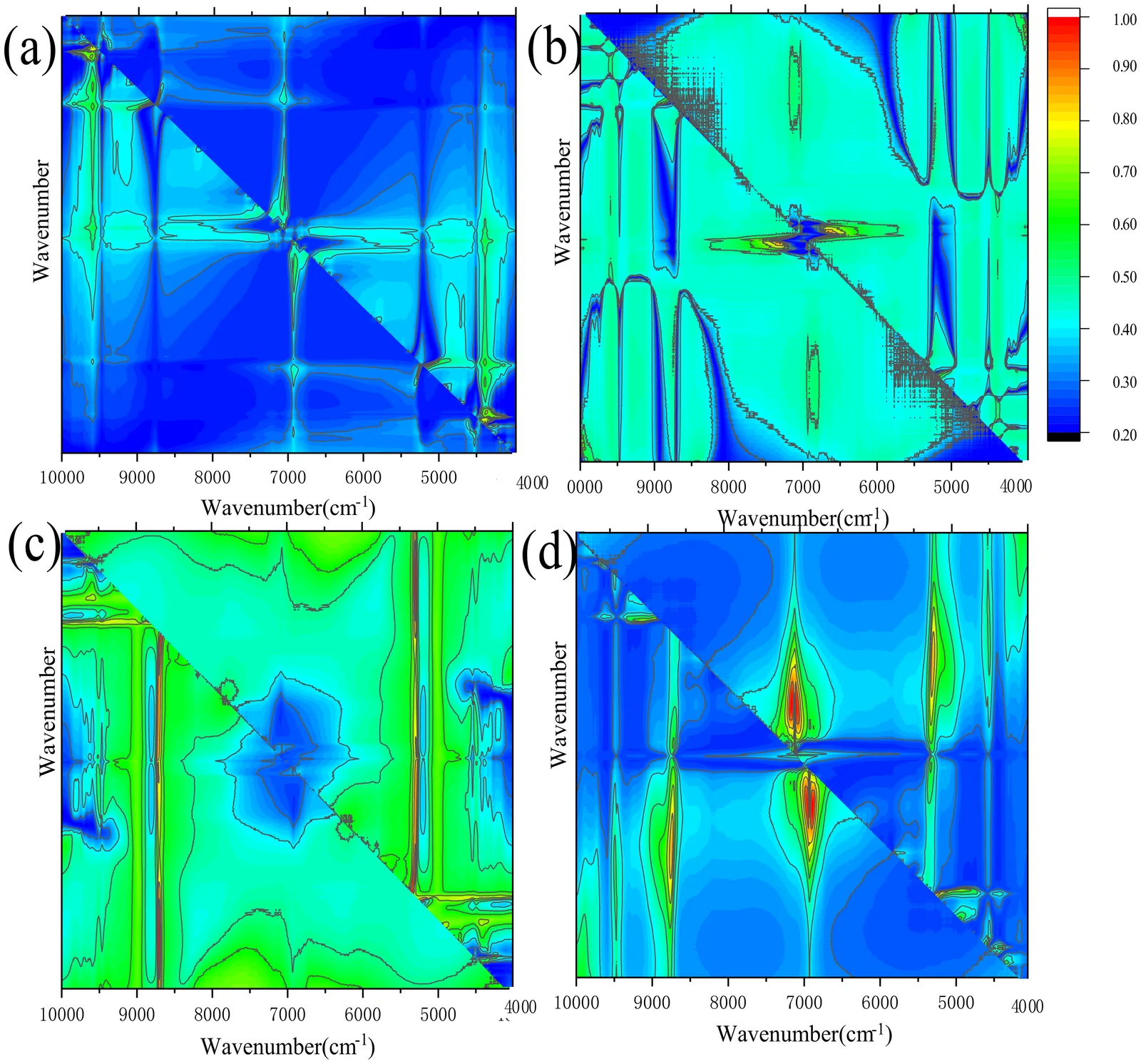
Correlation heatmaps between spectral wavenumbers (x-axis: wavenumber; y-axis: wavenumber) under different preprocessing strategies: **(a)** raw spectra, **(b)** MSC, **(c)** SNV, and **(d)** SG smoothing.

To further validate the effectiveness of different spectral preprocessing strategies in regression modeling, four commonly used models PLSR, KNN, RF and 1D-Res-CNN were employed using spectra preprocessed with MSC, SNV, and SG techniques. **Table 3** summarizes the modeling performance across training and test sets using key evaluation metrics, including RMSECV, RMSEP, and the $R_{p}^{2}$. Overall, the SNV method demonstrated superior or near-optimal predictive performance across all three models. In the PLSR model, SNV-preprocessed spectra yielded the lowest RMSECV and RMSEP values, with an $R_{p}^{2}$ value of 0.93, outperforming the results obtained using raw, unprocessed spectra. The KNN and RF model, known for its sensitivity to data scaling and variability, showed suboptimal performance with raw spectra. While SNV did not achieve the absolute lowest RMSEP or highest $R_{p}^{2}$ in this case, it notably improved both the accuracy and stability of the model, indicating that SNV preprocessing is also well-suited for nonlinear algorithms. Similar trends were observed in the 1D-Res-CNN model. While MSC and SG offered only marginal improvements, SNV preprocessing achieved an $R_{p}^{2}$ of 0.93 and RMSEP of 2.15, leading to a further enhancement in predictive accuracy. These results underscore the robustness and consistency of SNV across both traditional and deep learning modeling frameworks.

**Table 3:** Cross-model comparison of prediction accuracy for soil organic matter (10000–4000 cm^-1^ interval).

Model	Preprocessing method	Train dataset		Test dataset	
		RMSECV	$R_{\mathrm{CV}}^{2}$	RMSEP	$R_{p}^{2}$
PLSR	RAW	2.76	0.89	2.72	0.90
	MSC	6.23	0.70	6.31	0.70
	SNV	1.73	0.95	2.20	0.93
	SG	3.09	0.86	3.85	0.78
KNN	RAW	6.23	0.68	5.91	0.74
	MSC	5.86	0.72	5.41	0.76
	SNV	4.88	0.78	4.32	0.80
	SG	4.12	0.80	3.97	0.82
RF	RAW	5.12	0.74	5.43	0.72
	MSC	4.56	0.78	5.41	0.77
	SNV	3.88	0.82	4.02	0.82
	SG	3.98	0.82	4.56	0.81
1D-Res-CNN	RAW	2.01	0.95	2.17	0.93
	MSC	3.67	0.83	3.82	0.82
	SNV	2.17	0.96	2.15	0.93
	SG	2.86	0.85	2.92	0.84

Overall, the SNV method effectively mitigates baseline shifts and scale inconsistencies caused by scattering path length variations in field-scale agricultural soils. From both a mechanistic perspective and a modelling standpoint, SNV offers substantial practical value, particularly for heterogeneous or granular soils. It enhances the signal-to-noise ratio in spectrally sensitive regions, amplifies the representation of key variables, and demonstrates robust adaptability across both conventional statistical models and deep learning frameworks. The findings of this study suggest that SNV serves as a highly suitable preprocessing strategy, capable of producing a noise-reduced and scale-consistent input space for subsequent multivariate regression tasks. The final improvement enhances the prediction accuracy of pH, labile organic carbon, total nitrogen, available phosphorus, and organic matter content in field-scale agricultural soils.

### 3.4 Mechanistic insights from 1D-Res-CNN feature visualization

Compared to PLSR, RF and KNN, the 1D-Res-CNN model demonstrates significant advantages in predicting soil chemical constituents. To further explore and maximize the potential of 1D-Res-CNN in spectral analysis, this study investigates the feature learning process of the model by visualizing the intermediate feature maps produced during convolutional operations. The proposed 1D-Res-CNN architecture consists of three convolutional layers. By extracting and visualizing the feature maps from each layer, the progressive transformation of spectral features through the network can be intuitively observed. The feature maps presented in **Figure 6** were extracted from the 1D-Res-CNN trained for soil organic matter prediction (selected as the representative property). To ensure robustness, the displayed maps represent the mean activation across 100 randomly selected calibration samples. Activations are plotted in their raw post-ReLU values without additional scaling or normalization.

**Figure 6:**
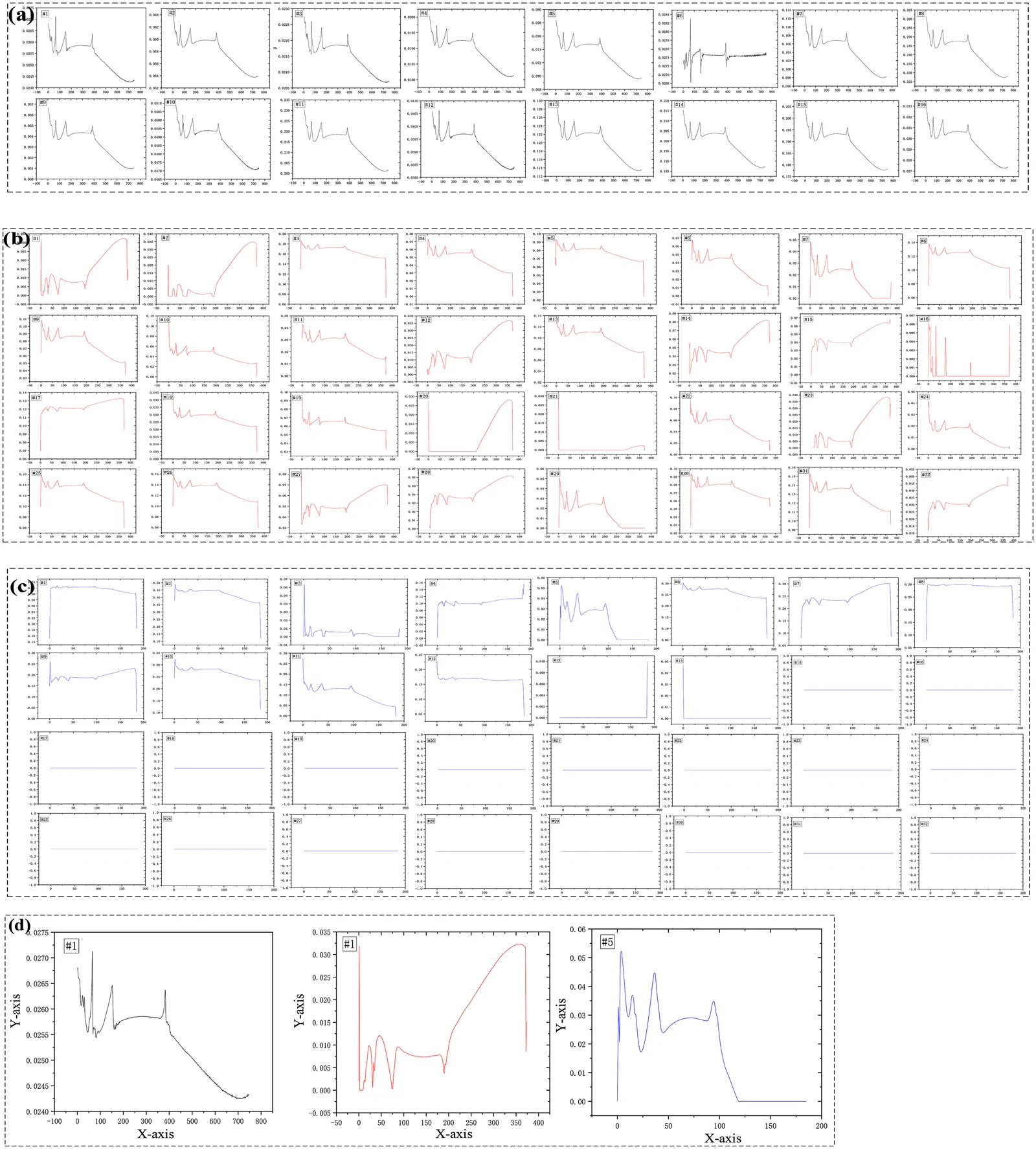
Output feature maps of the 1D-Res-CNN. **(a)** Layer 1 (16 maps): initial spectral responses. **(b)** Layer 2 (32 maps): abstracted features. **(c)** Layer 3 (64 maps, top 16 non-zero activations shown). **(d)** Enlarged representative maps from each layer with axis labels; x-axis: spectral position, y-axis: activation magnitude (post-ReLU).

The first convolutional block comprises 16 kernels. Convolving the one-dimensional spectral input with these kernels yields 16 feature maps. The panel indices label individual channels, the abscissa corresponds to wavenumber (cm^-1^), and the ordinate represents activation magnitude. These maps reveal, layer by layer, how the 1D-Res-CNN distils spectral information. To quantitatively assess the contribution of specific spectral regions to the final prediction, a wavenumber (cm^-1^) occlusion sensitivity analysis was performed. Sequential 50-cm^-1^ spectral windows were masked (set to the mean spectrum value) in the prediction set, and the resulting increase in RMSEP was recorded. Spectral regions exhibiting the largest RMSEP increment upon occlusion were identified as the most influential for model prediction. The first-layer feature maps exhibited activation peaks spatially aligned with known functional-group absorption regions. While this spatial alignment is consistent with physically meaningful spectral feature extraction, we acknowledge that visual similarity alone does not establish causal contribution to the final prediction.

The 16 feature maps generated by the first convolutional layer are forwarded to the second layer, where 32 additional feature maps are produced. As the network deepens, each successive convolution extracts increasingly abstract representations while reducing spatial dimensionality, thereby enhancing salient spectral features and suppressing irrelevant noise. The second convolutional layer notably emphasises NIR absorption characteristics: approximately 90% of its kernels exhibit strong activations within the 10,000~4,000 cm^-1^ range, reflecting heightened sensitivity to chemically informative bands. In contrast, kernels 17 and 18 respond primarily to localized spectral fluctuations; their feature maps show low-amplitude variations and minimal activations. By comparison, kernels 4 through 9 capture the global spectral profile, retaining the overall waveform shape while accentuating key absorption peaks—thereby playing a pivotal role in enhancing prediction accuracy. The third convolutional layer employs 64 convolutional kernels. To assess whether the near-zero activation phenomenon is consistent across the dataset, we quantified the sparsity across the entire calibration set. The proportion of near-zero activations in the third layer was 75%, confirming that this is a systematic property rather than an artifact of a single sample. Retained the 16 channels with the highest mean activation magnitude for visualization, while omitting the remaining 48 channels due to their negligible response. This high sparsity in deep layers is consistent with the behavior of hierarchical feature extractors: as the network depth increases, individual kernels specialize in highly specific spectral patterns, leading to selective activation on only a subset of samples or spectral regions. It does not necessarily indicate channel redundancy or over-parameterization; rather, it reflects the adaptive compression of spectral information into a compact set of discriminative feature detectors. To demonstrate the readability of axis labels at publication scale, one representative feature map from each convolutional layer (Layer 1: #1, Layer 2: #1, Layer 3: #5) is enlarged in panel (d), with the abscissa representing spectral position (down-sampled wavelength index) and the ordinate representing activation magnitude (post-ReLU output value).

### 3.5 Comparative evaluation of GASF and CWT encoding

Generating spectrograms through Gramian Angular Field and Continuous Wavelet Transform preserves the full spectral signature, a prerequisite for accurate quantification of soil chemical constituents. **Figure 7** illustrates the spectrograms produced by Continuous Wavelet Transform across the three spectral sub-ranges. In this study, the entire spectral range was divided into two segments: 10000~5880 cm^-1^ and 5880~4000 cm^-1^. The frequency of 5880 cm^-1^ is a specific point in the infrared spectrum. The boundary at 5880 cm^-1^ was selected as an empirical partition point based on the observed spectral characteristics of the dataset. The region below 5880 cm^-1^ contains the majority of the prominent absorption features in the acquired FT-NIR spectra (**Figure 4**), whereas the region above 5880 cm^-1^ exhibits relatively smooth baseline-like variation with fewer distinct peaks. This boundary does not correspond to a universally accepted physical distinction between skeletal and functional-group vibrations in the NIR region, as NIR spectra are dominated by overtone and combination bands rather than fundamental vibrations. **Figure 8** displays the spectrograms generated by the Gramian Angular Field approach. (**Figure 8a**) depicts the transformation of the spectrum within the 5880~4000 cm^-1^ range into a spectrogram, (**Figure 8b**) shows the transformation of the spectrum within the 10000~5880 cm^-1^ range, and (**Figure 8c**) illustrates the transformation of the entire 10000~4000 cm^-1^ range. The left panel represents the original spectral sequence, the middle panel shows the corresponding polar coordinate projection, and the right panel displays the final two-dimensional GASF-encoded image. Initially, the one-dimensional spectral sequence is mapped onto the polar coordinate space and then further converted into a two-dimensional image using the GASF method. Compared with the traditional Cartesian coordinate system, the polar coordinate system is more effective in preserving the dependencies within the spectral sequence. After normalizing the spectral data and projecting it onto the polar coordinate plane, the geometric features of each sample in terms of angle and radius reflect the changes in local absorption intensity. Due to the geometric transformation in polar coordinates, the angles corresponding to different spectral ranges change, resulting in significant differences in the polar angle distribution of samples across various bands. This characteristic ensures that the polar coordinate encoding results of each sample in the two spectral segments are distinct, providing a robust foundation for subsequent image classification models to capture fine-grained differences.

**Figure 7:**
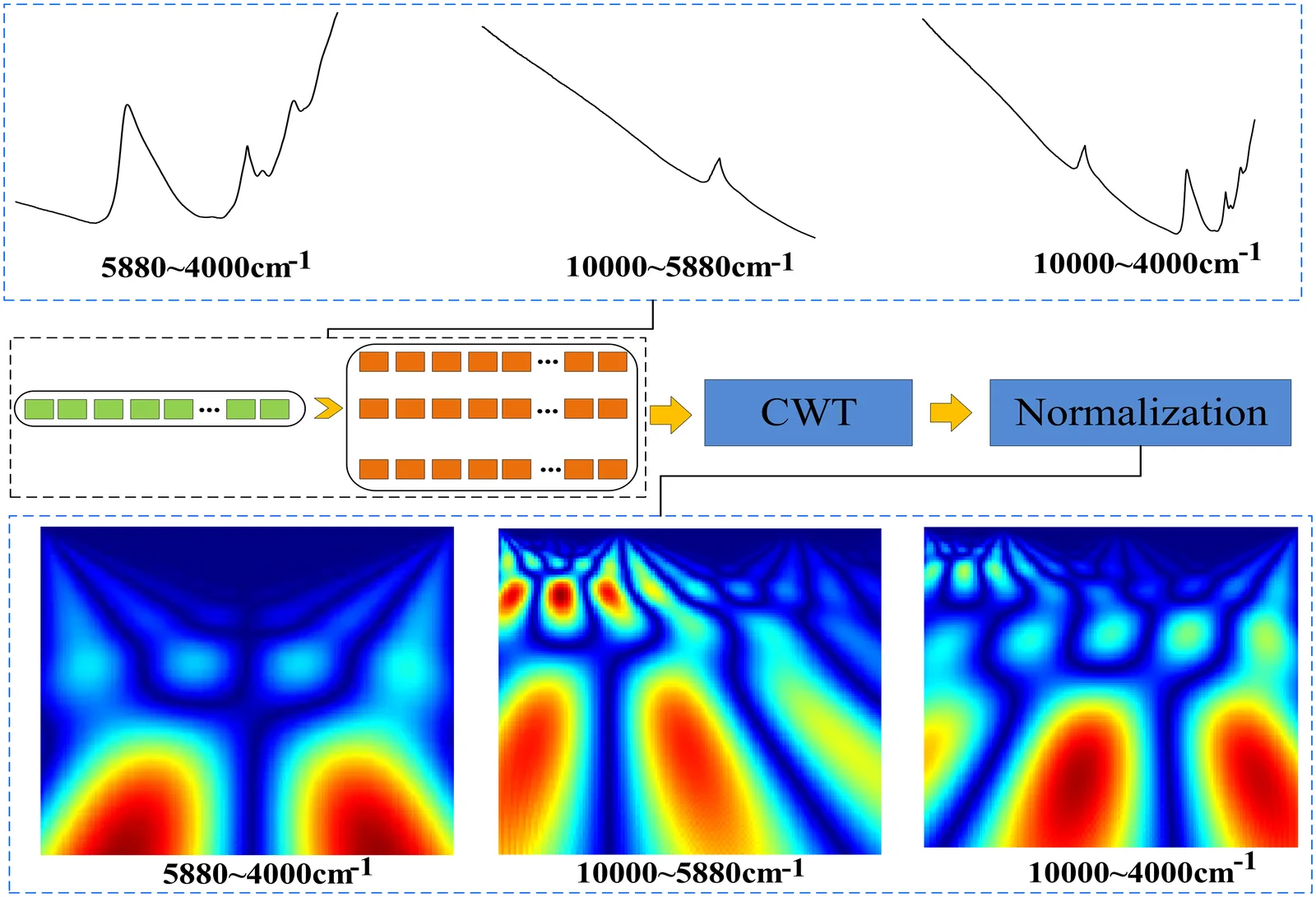
Spectrogram evolution under the continuous wavelet transform pipeline.

**Figure 8:**
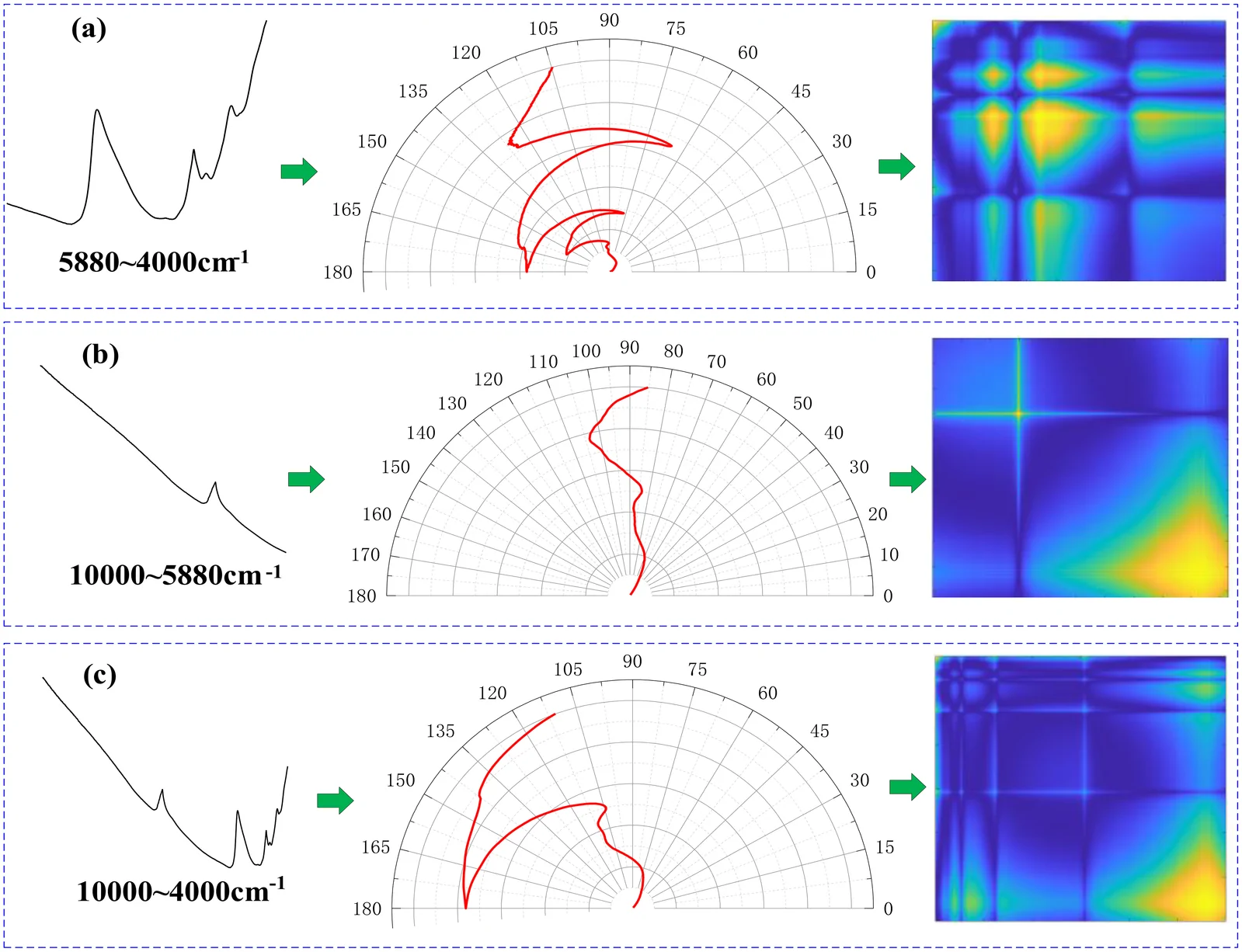
The process of 2D spectrogram transformation. **(a)** 5880~4000 cm^-1^, **(b)** 10000~5880 cm^-1^, **(c)** 10000~4000 cm^-1^.

During visual encoding, the spectral vector is first projected into angular space via the arccosine function; pairwise angular cosines are then computed across the resulting angle matrix to yield a symmetric two-dimensional image. For visualization, the image employs a blue-to-bright-yellow gradient (−1 to +1), with spectral wavenumbers ordered from the upper-left to the lower-right diagonal. This ordering preserves both the global structure and local correlations of the original spectrum. The optimal spectral interval was determined through a two-step procedure: (1) visual screening of encoded image structural density to identify candidate intervals; (2) quantitative validation via 2D-Res-CNN cross-validation across all interval-encoding combinations. The 5880–4000 cm^-1^ interval with GASF encoding, which exhibited the highest feature density in the visual screening, was subsequently confirmed to yield the best predictive performance in the quantitative modeling. **Figures 7**, **8** reveal pronounced visual disparities among the three sub-ranges. In the 5880~4000 cm^-1^ segment, yellow high-value regions dominate, exhibiting three prominent peaks flanked by four distinct troughs—an indication of high spectral complexity and dense feature content. Conversely, the 10000~5880 cm^-1^ segment displays two gently intersecting bands, signifying smoother variability and lower information density. The full 10 000~4000 cm^-1^ image closely resembles its high-wave-number subset, implying that the predictive information for soil chemical constituents is concentrated below 5880 cm^-1^.

### 3.6 Prediction performance of the proposed 2D-Res-CNN

The full spectrum was partitioned into three sub-intervals (10000~5880 cm^-1^, 5880~4000 cm^-1^, and 10000~4000 cm^-1^), quantitative 2D-Res-CNN modeling was performed for all three intervals under both GASF and CWT encoding, the results are summarized in **Supplementary Table 4**. Cross-validation indicates that spectra encoded via Gramian Angular Field consistently yield higher coefficients of determination for every soil chemical constituent than those processed by Continuous Wavelet Transform, underscoring the superior predictive fidelity of the angular-encoding approach. The values of the training set for pH, labile organic carbon, total nitrogen, organic matter, and available phosphorus were all above 0.93. Specifically, the R^2^ for organic matter reached 0.99, and that for pH was 0.96, demonstrating strong separability of their spectral features. Cross-validation across all three spectral intervals and both encoding paradigms shows that the performance ranking is property-dependent. The 5880~4000 cm^-1^ interval with GAF encoding yielded the best prediction for total nitrogen and available phosphorus, and was essentially tied with full-spectrum GAF for labile organic carbon. For pH and organic matter, full-spectrum GAF gave marginally lower prediction errors; Overall, GAF encoding particularly in the 5880~4000 cm^-1^ interval performed best or statistically comparably for the majority of properties, which supports its adoption as the default encoding strategy. Notably, although the for available phosphorus was 0.96, its prediction set was 0.88, which was slightly lower than the prediction results for other chemical components. The RMSECV for available phosphorus was 5.87, and the RMSEP was 10.72, indicating less satisfactory model performance. This may be attributed to the complexity of phosphorus forms in soil and the weak specificity of their spectral absorption features. The prediction performance for organic matter, total nitrogen, and labile organic carbon was relatively strong, with prediction-set coefficients of determination of 0.93, 0.91, and 0.91, respectively. Importantly, the ranking of the candidate strategies was established from the outer loop of the nested cross-validation conducted within the calibration set: GAF-encoded spectrograms constructed from raw spectra, without any prior preprocessing, consistently outperformed the SNV-preprocessed one-dimensional spectra. And that for pH and available phosphorus reached 0.87 and 0.88, respectively. These results indicate that the GAF-based two-dimensional encoding of raw spectra yields improved predictive accuracy for most chemical constituents, underscoring the capability of the Gramian Angular Field method to enhance spectral feature representation (Feng et al., 2024). This conclusion follows a sequential control-of-variables logic: (i) under identical conditions (SNV preprocessing, full spectrum, and the same SPXY partitions), the 1D-Res-CNN outperformed PLSR, KNN, and RF (**Table 3**), establishing the contribution of the network architecture; (ii) applying visual encoding to raw full spectra improved the 2D-Res-CNN over the 1D benchmark for labile organic carbon, total nitrogen, and available phosphorus, with organic matter essentially tied and pH slightly lower, indicating that the encoding benefit is property-dependent; (iii) restricting the GAF-encoded input to the 5880~4000 cm^-1^ interval further improved total nitrogen, labile organic carbon, and available phosphorus relative to full-spectrum encoding (**Table 4**). For available phosphorus—the most weakly predicted property among the five—the largest absolute gain was obtained with GAF encoding, whose capability of reorganizing spectral structure may facilitate the unmixing of low-concentration characteristic peaks that are easily masked by water absorption bands and the broad spectra of organic matter. Overall, the combination of GASF and 2D-Res-CNN with residual connections not only identified the feature-rich spectral range of 5880~4000 cm^-1^, but also improved the prediction of chemical component contents in field-scale agricultural soils.

**Table 4:** Performance results of the 2D-Res-CNN with residual connections.

Spectral region	Chemical component categories	GASF				CWT			
		RMSECV	$R_{\mathrm{CV}}^{2}$	RMSEP	$R_{p}^{2}$	RMSECV	$R_{\mathrm{CV}}^{2}$	RMSEP	$R_{p}^{2}$
5880~4000 cm^-1^	pH	0.21	0.96	0.39	**0.87**	0.41	0.86	0.58	0.78
	Labile Organic Carbon	37.56	0.95	69.55	**0.91**	56.32	0.85	76.56	0.88
	Total Nitrogen	2.40	0.93	3.06	**0.91**	2.32	0.87	4.32	0.86
	Organic Matter	2.17	0.99	2.85	**0.93**	1.98	0.90	3.56	0.85
	Available phosphorus	5.87	0.96	10.72	**0.88**	4.32	0.91	12.23	0.84
10000~5880 cm^-1^	pH	0.41	0.88	0.53	0.78	0.39	0.86	0.62	0.79
	Labile Organic Carbon	98.78	0.81	111.43	0.76	74.55	0.90	97.74	0.81
	Total Nitrogen	3.24	0.85	4.31	0.75	4.33	0.82	5.01	0.78
	Organic Matter	3.86	0.88	5.18	0.78	6.79	0.67	7.22	0.67
	Available phosphorus	17.36	0.76	16.69	0.76	24.03	0.73	22.11	0.68
10000~4000 cm^-1^	pH	0.22	0.96	0.21	0.88	0.36	0.92	0.63	0.78
	Labile Organic Carbon	37.57	0.95	69.55	0.90	75.98	0.83	80.12	0.82
	Total Nitrogen	3.02	0.97	3.92	0.88	2.86	0.92	1.88	0.90
	Organic Matter	0.92	0.98	2.10	0.93	2.76	0.88	2.68	0.88
	Available phosphorus	5.96	0.96	18.73	0.82	6.87	0.90	8.02	0.89

Nc and Np denote the numbers of calibration and prediction samples, respectively. Sample sizes (Nc/Np): pH (1,756/439); labile organic carbon (1,412/352); total nitrogen (1,756/439); organic matter (1,729/433); available phosphorus (1,756/439). Units for RMSECV and RMSEP correspond to those listed in **Table 2**. Bold values indicate the best-performing encoding strategy for each soil property within each spectral region.

Scatter plots provide an intuitive visualization of the predicted and actual values of soil chemical component contents, revealing the distribution patterns of the data. If the scatter points closely follow a straight line with a slope of 1, it indicates that the predicted values are very close to the actual values, suggesting good model prediction performance. As shown in **Figure 9**, scatter plots of the 2D-Res-CNN predictions for soil chemical components are presented. The horizontal axis represents the actual values of the chemical component contents, while the vertical axis represents the predicted values. The pH prediction model results are shown in (**Figure 9a**), with 1756 samples in the training set and 439 samples in the test set. The determination coefficients for the training and test sets are 0.96 and 0.87, respectively, indicating strong predictive capability of the model. (**Figures 9b–e**) show the prediction results for labile organic carbon, total nitrogen, organic matter, and available phosphorus, respectively. The blue dots represent the training set, while the red dots represent the test set. The total number of samples for each of the five chemical components is approximately 2000. Using the SPXY algorithm, the training and test sets were divided in a 3:1 ratio for each chemical component. The determination coefficients for the test sets of labile organic carbon, total nitrogen, organic matter, and available phosphorus are 0.91, 0.91, 0.93, and 0.88, respectively. The 2D-Res-CNN model demonstrates good generalization ability for new data when predicting soil chemical component contents in field-scale agricultural soils, effectively capturing the intrinsic structure of the dataset. The specialized architecture of the 2D-Res-CNN, combined with the GASF module, enhances the model’s accuracy and efficiency, especially in datasets with a large number of variables. When the number of variables is relatively small, the advantages of the integrated model may not be fully realised, potentially leading to performance degradation. Overall, the GASF-integrated 2D-Res-CNN model outperforms PLSR and KNN models in predicting soil chemical component contents in field-scale agricultural soils (Ng et al., 2019). Given that PLSR and KNN models have demonstrated high accuracy on public datasets, this further underscores the broad application potential of the GASF-integrated 2D-Res-CNN as a tool for accurately predicting target components.

**Figure 9:**
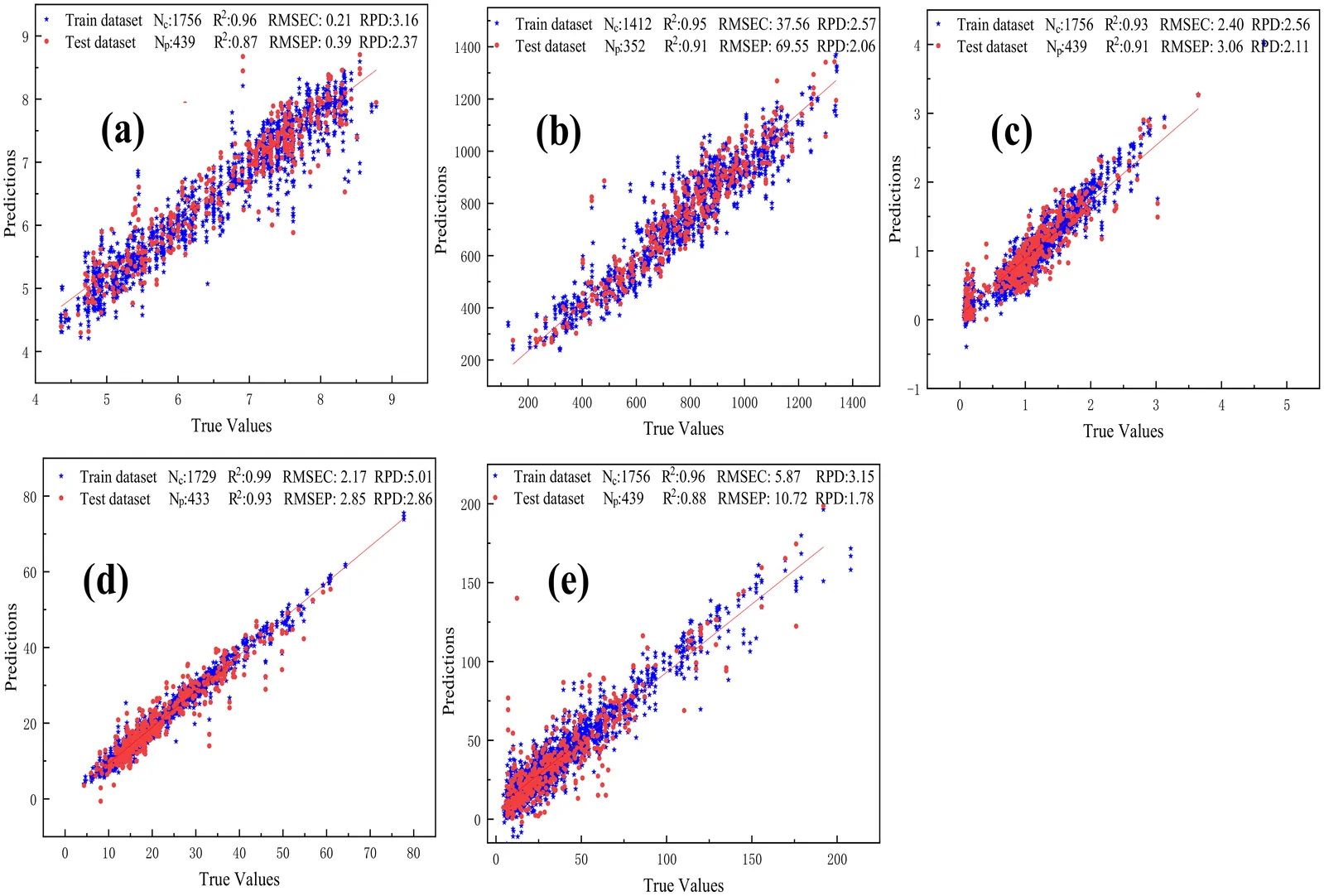
Scatter plots of the prediction results from the 2D-Res-CNN model: **(a)** pH; **(b)** Labile organic carbon; **(c)** Total nitrogen; **(d)** Organic matter; **(e)** Available phosphorus.

## 4 Conclusions

This study presents an interpretable deep-learning framework that couples visual encoding with residual-connected convolutional neural networks for soil chemical analysis. Intermediate-layer feature visualization of the 1D-Res-CNN reveals activation patterns that are qualitatively consistent with known functional-group absorption regions, offering preliminary insight into the model’s spectral learning behavior. Quantitative attribution analysis provides supporting evidence that the model leverages chemically informative bands, though acknowledged that this does not constitute definitive mechanistic interpretation. GASF and CWT were employed to recast one-dimensional spectral sequences into two-dimensional images; Quantitative comparison across three spectral intervals shows that GASF encoding—most notably within the 5880~4000 cm^-1^ interval yields the highest or statistically comparable predictive accuracy for the majority of the five properties. The proposed GASF-driven 2D-Res-CNN achieved prediction-set of 0.87, 0.91, 0.91, 0.93, and 0.88 for soil pH, labile organic carbon, total nitrogen, organic matter, and available phosphorus, respectively. Notably, available phosphorus prediction regarded as exploratory and suitable for preliminary screening only, pending stronger external validation. Without independent field-grouped validation, the reported results demonstrate internal prediction within the sampled dataset. Broad applicability to new agricultural soils remains to be established through future spatially independent validation campaigns. By enhancing spectral feature expressiveness through visual encoding and providing preliminary insights into model behavior via layer-wise visualization, this work offers a technical strategy for rapid soil chemical assessment. However, caution that claims regarding the resolution of the CNN “black box” or the achievement of definitive mechanistic interpretation require further validation through targeted spectroscopic experiments and larger spatially diverse datasets.

## Data Availability

The raw data supporting the conclusions of this article will be made available by the authors, without undue reservation.
